# iTRAQ-Based Proteomic Analysis of Polyploid Giant Cancer Cells and Budding Progeny Cells Reveals Several Distinct Pathways for Ovarian Cancer Development

**DOI:** 10.1371/journal.pone.0080120

**Published:** 2013-11-14

**Authors:** Shiwu Zhang, Imelda Mercado-Uribe, Samir Hanash, Jinsong Liu

**Affiliations:** 1 Department of Pathology, Tianjin Union Medicine Center (Nankai University Affiliated Hospital), Tianjin, People’s Republic of China; 2 Department of Pathology, The University of Texas MD Anderson Cancer Center, Houston, Texas, United States of America; 3 Department of Clinical Cancer Prevention, The University of Texas MD Anderson Cancer Center, Houston, Texas, United States of America; University of Miami School of Medicine, United States of America

## Abstract

Polyploid giant cancer cells (PGCCs) are a morphologically distinct subgroup of human tumor cells with increased nuclear size or multiple nuclei, but they are generally considered unimportant because they are presumed to be nondividing and thus nonviable. We have recently shown that these large cancer cells are not only viable but also can divide asymmetrically and yield progeny cancer cells with cancer stem-like properties via budding division. To further understand the molecular events involved in the regulation of PGCCs and the generation of their progeny cancer cells, we comparatively analyzed the proteomic profiles of PGCCs, PGCCs with budding daughter cells, and regular control cancer cells from the HEY and SKOv3 human ovarian cancer cell lines with and without CoCl_2_. We used a high-throughput iTRAQ-based proteomic methodology coupled with liquid chromatography-electrospray ionization tandem mass spectroscopy to determine the differentiated regulated proteins. We performed Western blotting and immunohistochemical analyses to validate the differences in the expression patterns of a variety of proteins between PGCCs or budding PGCCs and regular cancer cells identified by iTRAQ approach and also a selected group of proteins from the literature. The differentially regulated proteins included proteins involved in response to hypoxia, stem cell generation, chromatin remodeling, cell-cycle regulation, and invasion and metastasis. In particular, we found that HIF-1alpha and its known target STC1 are upregulated in PGCCs. In addition, we found that a panel of stem cell-regulating factors and epithelial-to-mesenchymal transition regulatory transcription factors were upregulated in budding PGCCs, whereas expression of the histone 1 family of nucleosomal linker proteins was consistently lower in PGCCs than in control cells. Thus, proteomic expression patterns provide valuable insight into the underlying mechanisms of PGCC formation and the relationship between PGCCs and cancer stem cells in patients with ovarian cancers.

## Introduction

Polyploid giant cancer cells (PGCCs) are a subset of large atypical cancer cells found mostly in solid tumors. PGCCs are the major contributor to epithelial tumor heterogeneity and comprise 0.1% to 20% of tumor volumes, with these percentages increasing with stage and malignancy [1,2]. The nuclear features of these large tumor cells, including their nuclear size and shape, chromatin pattern, number of nucleoli, and number of nuclei, are among the most commonly described histopathologic features of human tumors. The number of PGCCs usually increases with the pathologic grade and stage [3-5]. Our recent data demonstrated that PGCCs contribute to solid tumor heterogeneity and play an important role in tumor initiation, metastasis, and chemoresistance [6] and formation of erythroid cells from normal fibroblasts and cancer cells [7] . Certain antimitotic chemotherapy drugs can also increase the formation of PGCCs in tumors, and PGCCs are often considered to be at the stage of mitotic catastrophe and on the verge of apoptosis [8]. Polyploid giant cells can also be observed in skeletal muscles during normal growth, osteoclasts, virally infected cells, tissue cultures, aging (senescent) cells [9], and stressed (e.g., oxidative or metabolic stress) cells and can be generated via cell fusion or abortive cell cycles [10]. PGCCs can also revert to regular-sized cancer cells (diploid cancer cells) in a division process called deploidization [11-14] or neosis [15]. All of these features indicate that PGCCs may play an important role in tumor development. However, PGCCs have not attracted major attention in the cancer research community owing to their poorly understood biology in tumors.

It is well known that tumors grow in a hypoxic environment, and hypoxia can facilitate the formation and maintenance of cancer stem cells and thus stimulate tumor growth [16-18]. Recently, we used cobalt chloride (CoCl_2_), a hypoxia mimetic widely used to treat anemia [19,20], to purify and achieve stable growth of PGCCs that otherwise would have differentiated into regular-sized cancer cells and demonstrated that PGCCs have cancer stem cell-like properties [6]. To further understand the underlying mechanisms involved in the differential regulation of regular cancer cells, PGCCs, and PGCCs with budding daughter cells we employed isobaric tagging for relative and absolute quantitation (iTRAQ) to identify differentially expressed proteins in PGCCs and budding progeny cells using the HEY and SKOv3 cell lines as model systems. 

## Materials and Methods

### Ethics Statement

The care and use of the mice were approved by the MD Anderson Institutional Animal Care and Use Committee.

### Cancer cell lines and culture

The human ovarian cancer cell lines HEY and SKOv3 were purchased from the American Type Culture Collection. The culture of HEY and SKOv3 cells was described previously by our group [21]. The two cancer cell lines were maintained in complete Eagle’s minimum essential medium (EMEM), which is minimum essential medium supplemented with fetal bovine serum and antibiotics.

### Generation and purification of PGCCs

HEY and SKOv3 cells were cultured in complete EMEM in T75 flasks until they reached 90% confluence. For PGCC generation, CoCl_2_ (Sigma-Aldrich, St. Louis, MO, USA) was added to the flasks to a final concentration of 300 μM and cultured for 48-72 h, as described previously [6]. After being rinsed with 1× phosphate-buffered saline (PBS), the cells were cultured in regular EMEM. Most regular-sized HEY cells died following this treatment, and only scattered PGCCs survived after treatment with CoCl_2_. Ten to 14 days later, the PGCCs recovered from treatment with CoCl_2_ and budding daughter cells derived from PGCCs were observed and photographed. After three times of treatments with CoCl_2_ (to acquire enough purified PGCCs), flasks of purified PGCCs (1×10^6^) were harvested for Western blotting and iTRAQ analysis before the PGCCs generated daughter cells. When the PGCCs were cultured in complete medium and recovered from three or four treatments with CoCl_2_, PGCCs (4×10^4^) with newly budding daughter cells (1×10^5^) (approximately 30% PGCCs and 70% budding daughter cells) were used for protein extraction and later analysis. 

### Preparation of protein extracts

Fresh pellets of purified PGCCs, 30% recovered PGCCs and 70% small daughter cells, and control cells were resuspended in 1 mL of cell wash buffer (ProteaPrep Cell Lysis Kit; Protea Biosciences, Morgantown, WV, USA) and then centrifuged at 12,000 × *g* for 5 min at 4°C. After two washes, the cell pellets were resuspended in 500 μL of ProteaPrep cell lysis buffer and incubated on ice for 30 min. Intermittent sonication was used to fully lyse the cells; the cell lysate was centrifuged at 12,000 × *g* for 10 min at 4°C, and the supernatant was transferred to a clean 1.5-mL tube. Prior to storage of the supernatant at -70°C, anionic acid labile surfactant II detergent (Protea Biosciences) build-up on the supernatant was degraded using formic acid.

### iTRAQ labeling of protein samples

iTRAQ-based proteomic analysis was done by the Protea Biosciences (Please refer to the manufacture’s instructions). Briefly, after the concentrations of five samples of each protein analyzed were measured, the samples were precipitated with acetone at a 6:1 ratio (acetone:sample), and the total protein isolates of five samples were resuspended in less than 60 µL of dissolution buffer and 1 µL of denaturant from the iTRAQ kit. Then 2 µL of reducing agent and 1 µL of cysteine blocking reagent were added to each of the samples. After digestion by adding 2 µg of trypsin, the samples were labeled with iTRAQ reagents (Table S1) by adding the contents of the iTRAQ vial to the sample solutions (Protea Biosciences). The iTRAQ reagents were reconstituted with 50 µL of ethanol. After the iTRAQ vial was added, the samples were incubated at room temperature for 60 min. 100 µL of de-ionized water was added to each sample vial, and the samples were incubated for 30 min at room temperature. The samples to be compared with each other were combined in a group and were lyophilized and reconstituted in strong cation exchange (SCX) reconstitution buffer.

### High-performance liquid chromatography fractionation

The samples of protein were fractionated using SCX ProteaTip SpinTips (Protea Biosciences). The tips were first washed twice by adding 50 μL of SCX reconstitution solution (Protea Biosciences). The samples were then loaded in spin tips and centrifuged at 6000 rpm for 3 min and then washed by adding 50 µL of reconstitution solution to the top of the spin tip. The samples in the spin tips were then washed again with SCX reconstitution solution and eluted sequentially with 150 μL of elution solution composed of 20, 40, 60, 80, 100, 150, 250, or 500 mM ammonium formate in 10% acetonitrile. Eight fractions corresponding to each salt concentration were collected. Each fraction was cleaned by repetitive lyophilization and acid treatment. After final lyophilization, the digests were reconstituted in 40 µL of acetonitrile/water/formic acid (5%/95%/0.1%)

### Liquid chromatography-electrospray ionization mass spectrometry

Mass spectrometric (MS) analysis of these samples was performed using a QTRAP 5500 system (Applied Biosystems, Toronto, Canada) for the acquisition of MS and tandem MS (MS/MS) data. Peptides were loaded on a Kinetex 100.0 × 2.1-mm C18 column (100 Å, 2.6 μm; Phenomenex, Torrance, CA, USA) and then submitted to mobile-phase elution in buffer A and buffer B (see Table S2 for detailed information on elution). The peptides were eluted at a flow rate of 200 μL/min. The liquid chromatography eluent was directed to an electrospray ionization source for quantitative time-of-flight MS analysis. Electrospray ionization was performed for information-dependent acquisition in the positive-ion mode with a spray voltage of 5 kV and selected mass range of 100-1000 *m*/*z*. The QTRAP 5500 system was operated in data-dependent acquisition mode. The three most abundantly charged peptides above a 5-count threshold were selected for MS/MS. 

Peptides were identified and quantified using ABI Protein ProteinPilot software 3.0 (Applied Biosystems, CA, USA). The Paragon algorithm in the ProteinPilot software was used for the peptide identification. Each MS/MS spectrum was searched against the International Protein Index human protein database, and the identified proteins with 95% confidence were accepted on the basis of their confidence scores obtained using the ProteinPilot software. Percent coverage was calculated as the percentage of matching amino acids from identified peptides having confidence greater than 0 divided by the total number of amino acids in the sequence.

### Immunohistochemical staining of PGCC-derived tumors in mice

The inoculation of nude mice with regular HEY cancer cells or PGCCs and subsequent tumor growth were described previously [6]. Ten PGCCs and 1×10^6^ regular HEY cancer cells for each nude mice were subcutaneously injected into the flanks of mice. The mice were killed and the tumors removed when the average tumor diameter reached 0.5–1.0 cm. Immunohistochemical staining of the tumor tissue was performed using the avidin-biotin-peroxidase method as described previously [6]. Paraffin-embedded tumor tissue was deparaffinized in xylene and rehydrated using graded dilutions of alcohol into water. After being washed with PBS, sections of the tumor tissue were subjected to antigen retrieval in 0.01 M sodium citrate buffer (pH 6.0) in an autoclave for 10 min. After endogenous peroxidase activity and nonspecific protein binding in the sections were blocked, the sections were incubated with primary antibodies overnight at 4°C in a humidified chamber (see Table S3 for detailed antibody information). We subsequently treated the samples with a biotinylated goat anti-rabbit IgG, and the signal was detected using a labeled streptavidin-biotin system in the presence of the chromogen 3,3'-diaminobenzidine. The nuclei were counterstained with hematoxylin.

### Western blot analysis

Based on the proteomic results, a group of potentially important proteins selected from the literature were determined by Western blot. Cell extracts of purified PGCCs, PGCCs with budding daughter cells, and control cells were lysed in an ice-cold buffer solution. Proteins in the cells were separated on a 10% sodium dodecyl sulfate-polyacrylamide gel and transferred to a polyvinylidine fluoride membrane (GE Healthcare, Waukesha, WI, USA). After being blocked with 5% nonfat milk in Tris-buffered saline with 0.1% Tween-20 for 1 h at room temperature, the membranes were incubated with the appropriate primary antibodies at 4°C overnight and then with secondary antibodies at room temperature for 1 h. Protein expression was measured using premixed ECL Plus reagent (GE Healthcare Life Sciences, Pittsburgh, PA, USA) and developed using an X-OMAT 2000 film processor (Eastman Kodak, Rochester, NY, USA). All Western blot experiments were duplicated, and β-actin was used as a protein-loading control. 

## Results

### CoCl_2_-induced formation of PGCCs

**Figure 1:**
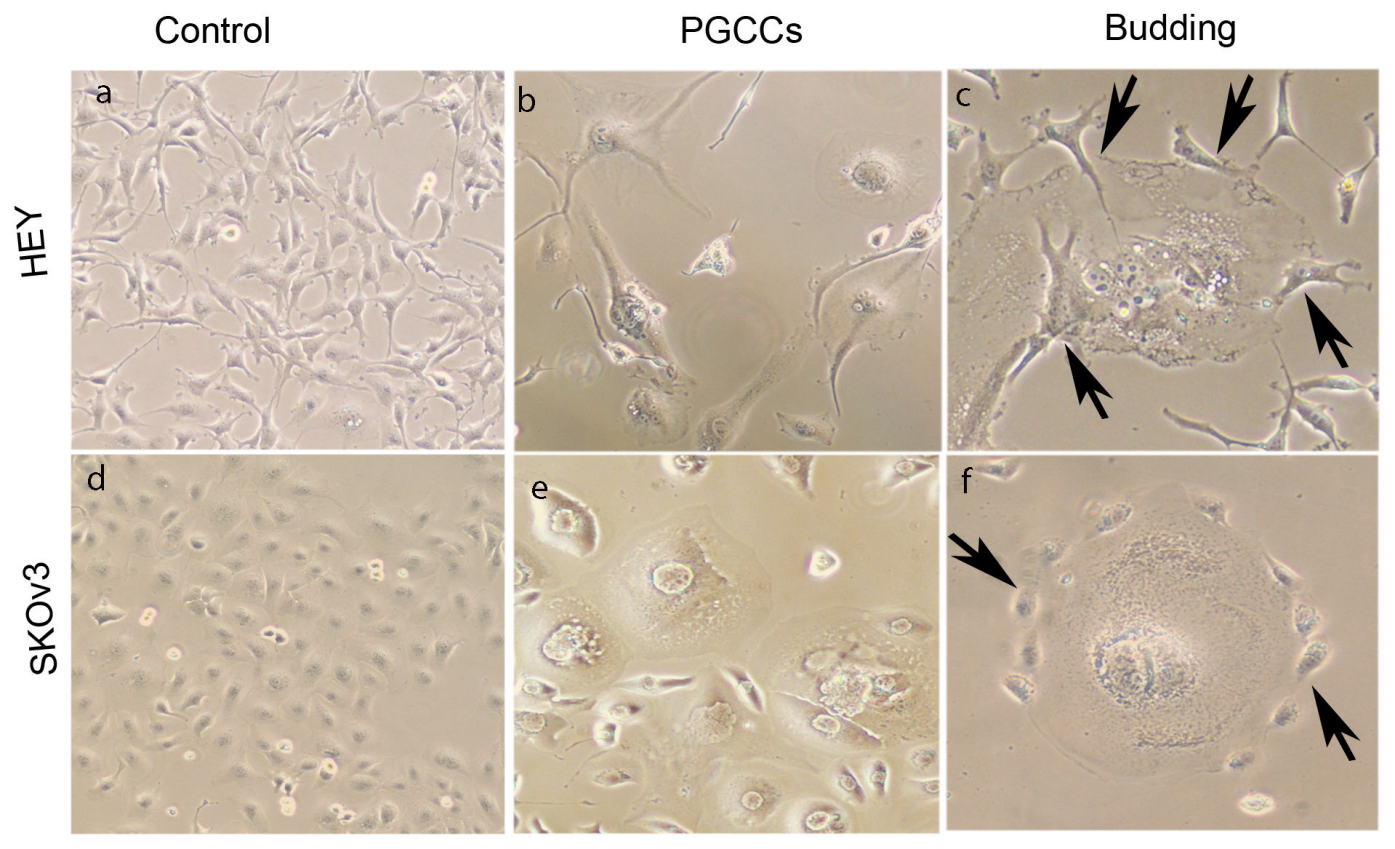
Control ovarian cancer cells, PGCCs alone, and PGCCs generating daughter cells via budding (10×). (A) Control HEY cells. (B) HEY PGCCs after treatment with CoCl_2_. (C) HEY PGCCs generating daughter cells via budding (black arrows). (D) Control SKOv3 cells. (E) SKOv3 PGCCs after treatment with CoCl_2_. (F) SKOv3 PGCCs generating daughter cells via budding (black arrows).

As we described previously, formation of PGCCs can be induced by treatment with CoCl_2_ [6]. High concentrations of CoCl_2_ selectively kill diploid cells, whereas low concentrations can induce PGCC formation via cell fusion. As shown in Figure 1, control HEY cells were irregular in shape with small apophyses. Treatment with CoCl_2_ at a high concentration (300 μM for 72 and 48 h for HEY and SKOv3 cells, respectively) killed most of the differentiated cells, and PGCCs could be clearly visualized after we removed any floating dead cells. After the cultures recovered from CoCl_2_ treatment, the surviving PGCCs generated daughter cells via budding when cultured in complete media with 10% serum [6]. 

### Protein identification and relative protein quantification in PGCCs and control cancer cells

To determine the global protein signature associated with the formation of PGCCs, we performed proteomic analysis of purified PGCCs and compared the protein expression in them with those in control cells and PGCCs undergoing budding. We isolated total protein extracts from HEY PGCCs alone, HEY PGCCs with budding daughter cells, control HEY cells, SKOv3 PGCCs alone, and control SKOv3 cells. Proteins were digested with trypsin, labeled with iTRAQ reagents (114-117), and analyzed using tandem mass spectrometry to identify differences in protein levels between these groups. To increase the coverage of protein identification and/or the confidence in the data generated, samples were iTRAQ-labeled in duplicate as follows: HEY PGCCs, 114; HEY PGCCs with budding, 115; control HEY, 116; SKOv3 PGCCs, 116; and control SKOv3, 117. Using iTRAQ, we identified and sorted the different proteins according to Unused ProtScore. The ratio of different iTRAQ labeling reagents indicated the relative abundance of the proteins. A total of 1,188 unique proteins were identified with 95% confidence by the ProteinPilot search algorithm and matched with proteins in the International Protein Index human protein database. Relative quantification of peptide was performed with ProteinPilot 3.0 software with statistical analysis (*P* < 0.05) for the selection of potentially differentially expressed proteins. In HEY PGCCs, 64 proteins were differently expressed when compared to PGCCs with budding daughter cells and control cells; 25 proteins were up-regulated in PGCCs and 39 were downregulated (Table 1). In SKOv3 PGCCs, 62 proteins were differently expressed when compared to control cells, with 40 up-regulated and 22 down-regulated (Table 2). The iTRAQ data from the various cell types of HEY could be classified into nine functional categories using the PANTHER classification system (www.pantherdb.org; Figure S1) including binding, catalytic activity, enzyme regulator activity, ion channel activity, motor activity, structural molecule activity, transcription regulator activity, translation regulator activity and transporter activity.

**Table 1:** Proteins Differentially Expressed Among HEY PGCCs, HEY PGCCs with Budding, and Control HEY.

Accession	Protein Name	Function	Peptides (95%)	% Coverage	Ratio (Sample 1:3)	P value (Sample 1:3	Ratio (Sample 2:3)	P value (Sample 2:3)
Upregulated (25)								
IPI00909303.2	CTSB cDNA FLJ58073, moderately similar to Cathepsin B(*)	Lysosomal cysteine protease	2	40	4.70	1.75E-02	4.56	1.46E-02
IPI00011229.1	CTSD Cathepsin D	Tumor invasiveness	1	32.77	4.16	5.22E-03	1.77	8.00E-02
IPI00183695.9	S100A10 Protein S100-A10	cell cycle progression and differentiation	1	48.45	3.38	6.16E-02	4.29	4.91E-02
IPI00845339.1	HSPA1B;HSPA1A cDNA FLJ54392, highly similar to Heat shock 70 kDa protein 1 (*)	protein folding, and help to protect cells from stress	8	49.45	3.26	7.62E-06	1.44	3.54E-02
IPI00737171.1	LOC729317 similar to voltage-dependent anion channel 2	Mitochondria-mediated apoptosis	4	21.84	2.71	8.39E-04	2.65	8.23E-02
IPI00216308.5	VDAC1 Voltage-dependent anion-selective channel protein 1	Mitochondria-mediated apoptosis	1	29.33	2.53	8.72E-02	1.68	1.73E-02
IPI00220827.5	TMSB10 Thymosin beta-10	Cytoskeleton	4	79.55	2.15	4.82E-01	2.61	1.33E-02
IPI00010402.2	SH3BGRL3 Putative uncharacterized protein	potentially involved in resistance of cells to the apoptosis-induced by TNF-α	4	58.41	2.14	1.36E-05	2.19	1.52E-03
IPI00186711.4	PLEC Isoform 2 of Plectin(*)	A link between the three main components of the cytoskeleton	4	33.67	2.04	2.02E-03	1.78	1.81E-02
IPI00020984.2	CANX cDNA FLJ55574, highly similar to Calnexin	Chaperone molecules (assisting protein folding and quality control)	2	31.90	1.98	1.38E-01	2.42	4.26E-02
IPI00940656.1	LOC723972;ANP32A 28 kDa protein	Inhibitor 1 of PP2A	4	43.72	1.90	2.36E-03	1.85	1.55E-02
IPI00872814.1	MSN (moesin) 68 kDa protein	Cross-linkers between plasma membranes and actin-based cytoskeletons	4	43.06	1.89	2.20E-04	1.79	4.65E-02
IPI00025491.1	EIF4A1 Eukaryotic initiation factor 4A-I	Protein synthesis	2	25.37	1.64	5.89E-03	0.96	9.17E-01
IPI00220362.5	HSPE1 10 kDa heat shock protein, mitochondrial(*)	Molecular chaperones	10	81.37	1.60	1.84E-02	0.97	9.53E-01
IPI00019502.3	MYH9 Isoform 1 of Myosin-9(*)	Muscle contraction and eukaryotic motility	2	33.52	1.57	4.19E-02	1.27	4.70E-01
IPI00795408.1	RPL23 15 kDa protein	Ribosomal protein	3	55.71	1.52	1.68E-02	0.89	5.64E-01
IPI00012442.1	G3BP1 Ras GTPase-activating protein-binding protein 1	RNA-binding proteins	3	33.48	1.50	3.45E-02	1.05	8.64E-01
IPI00018352.1	UCHL1 Ubiquitin carboxyl-terminal hydrolase isozyme L1	Protein post-translational modification	2	60.09	1.43	4.29E-02	1.15	3.60E-02
IPI00789551.1	SNHG4;MATR3 Putative uncharacterized protein MATR3	Interaction with nuclear matrix proteins to form the fibrogranular network	4	31.84	1.26	7.42E-01	1.45	4.94E-02
IPI00021405.3	LMNA Isoform A of Prelamin-A/C	Reassembly of the nuclear envelope and cell division	15	70.48	1.24	7.53E-03	1.05	7.28E-01
IPI00294578.1	TGM2 Isoform 1 of Protein-glutamine gamma-glutamyltransferase 2	absorption and secretion	3	21.40	1.24	9.07E-03	1.21	3.24E-01
IPI00021812.2	AHNAK Neuroblast differentiation-associated protein AHNAK(*)	The pseudopod protrusion and cell migration	90	84.77	1.20	4.81E-02	1.43	1.18E-02
IPI00099550.1	UBQLN1 Isoform 1 of Ubiquilin-1	Protein post-translational modification	2	22.24	1.18	9.96E-02	0.66	4.57E-02
IPI00916861.1	MDH1 Putative uncharacterized protein MDH1	An enzyme that catalyzes the oxidation of malate	1	34.32	1.11	5.28E-01	0.66	8.16E-03
IPI00927101.1	RPSA;RPSAP15;SNORA62;SNORA6 Putative uncharacterized protein RPSA	Non-integrin family, laminin receptor 1.	2	31.82	1.07	3.88E-01	0.40	1.30E-02
Downregulated (39)								
IPI00217030.10	RPS4X 40S ribosomal protein S4, X isoform	S4E family of ribosomal proteins.	1	50.19	0.98	8.27E-01	0.43	4.20E-02
IPI00003362.2	HSPA5 HSPA5 protein	Molecular chaperones	14	54.20	0.95	5.95E-01	0.77	4.22E-02
IPI00554788.5	KRT18 Keratin, type I cytoskeletal 18	A keratin protein related with secretory epithelia	8	53.26	0.88	3.80E-02	0.79	1.58E-01
IPI00003865.1	HSPA8 Isoform 1 of Heat shock cognate 71 kDa protein(*)	Molecular chaperones and as an ATPase in the disassembly of clathrin-coated vesicles	11	56.66	0.84	3.24E-01	0.44	8.60E-04
IPI00646304.4	PPIB Peptidyl-prolyl cis-trans isomerase B	Apoptotic and necrotic cell death	2	55.56	0.83	2.00E-01	0.49	1.41E-03
IPI00396485.3	EEF1A1 Elongation factor 1-alpha 1(*)	Translation and nuclear export of proteins	11	57.36	0.81	1.24E-01	0.40	5.84E-03
IPI00010204.1	SRSF3 Serine/arginine-rich splicing factor 3	RNA splicing	2	62.20	0.81	3.40E-02	0.75	6.19E-01
IPI00909232.1	HNRNPC cDNA FLJ53542, highly similar to Heterogeneous nuclear ribonucleoprotein C	Transcription	5	66.32	0.79	8.86E-02	0.54	1.71E-02
IPI00472119.2	- 30 kDa protein	Other	4	50.00	0.75	1.33E-02	0.58	2.80E-02
IPI00025252.1	PDIA3 Protein disulfide-isomerase A3	Other	3	30.10	0.75	3.20E-01	0.67	3.85E-02
IPI00290460.3	EIF3G Eukaryotic translation initiation factor 3 subunit G	Protein synthesis	1	18.44	0.75	1.95E-02	0.44	2.14E-01
IPI00383296.5	HNRNPM Isoform 2 of Heterogeneous nuclear ribonucleoprotein M	Protein synthesis	6	61.22	0.74	1.81E-03	0.67	4.79E-02
IPI00784154.1	HSPD1 60 kDa heat shock protein, mitochondrial	Molecular chaperones	13	56.20	0.72	3.32E-02	0.60	7.56E-02
IPI00216691.5	PFN1 Profilin-1	Cell motility	10	92.14	0.72	1.06E-01	0.41	4.52E-02
IPI00465248.5	ENO1 Isoform alpha-enolase of Alpha-enolase	Other	22	74.42	0.71	1.51E-04	0.41	8.15E-03
IPI00910458.1	HNRNPK cDNA FLJ54552, highly similar to Heterogeneous nuclear ribonucleoprotein K	Protein synthesis	6	65.83	0.69	9.61E-02	0.46	1.39E-02
IPI00479191.2	HNRNPH1 51 kDa protein	Other	5	48.73	0.69	1.38E-01	0.23	4.10E-02
IPI00554648.3	KRT8 Keratin, type II cytoskeletal 8	Cell skeletal	6	55.69	0.64	7.05E-02	0.35	2.24E-03
IPI00382470.3	HSP90AA1 Isoform 2 of Heat shock protein HSP 90-alpha	Molecular chaperones	9	41.22	0.62	1.20E-01	0.58	2.89E-02
IPI00215780.5	RPS19 40S ribosomal protein S19	Protein synthesis	5	62.07	0.60	2.58E-02	0.27	4.04E-02
IPI00020599.1	CALR Calreticulin	Promoting macrophages to engulf hazardous cancerous cells	3	31.65	0.58	4.41E-04	0.83	5.25E-01
IPI00008524.1	PABPC1 Isoform 1 of Polyadenylate-binding protein 1	Translation initiation	5	33.65	0.54	7.06E-04	0.51	7.02E-02
IPI00010740.1	SFPQ Isoform Long of Splicing factor, proline- and glutamine-rich	Protein synthesis	3	47.81	0.54	2.56E-03	0.48	1.33E-01
IPI00005087.1	TMOD3 Tropomodulin-3		1	27.27	0.54	9.94E-03	1.10	6.73E-01
IPI00012493.1	RPS20 40S ribosomal protein S20	Protein synthesis	1	47.90	0.53	4.10E-02	0.42	2.01E-01
IPI00465365.4	HNRNPA1 Isoform A1-A of Heterogeneous nuclear ribonucleoprotein A1	Protein synthesis	12	81.56	0.51	6.02E-04	0.46	3.10E-01
IPI00010779.4	TPM4 Isoform 1 of Tropomyosin alpha-4 chain	Cell motility	8	87.50	0.47	1.42E-02	1.59	1.75E-01
IPI00027834.3	HNRNPL Heterogeneous nuclear ribonucleoprotein L	Protein synthesis	1	29.71	0.47	4.74E-02	0.57	2.79E-01
IPI00396378.3	HNRNPA2B1 Isoform B1 of Heterogeneous nuclear ribonucleoproteins A2/B1	Protein synthesis	12	71.39	0.46	7.20E-03	0.39	2.49E-03
IPI00419585.9	PPIA Peptidyl-prolyl cis-trans isomerase A	Apoptotic and necrotic cell death	9	92.73	0.46	4.23E-03	0.37	2.04E-02
IPI00796366.2	MYL6 cDNA FLJ56329, highly similar to Myosin light polypeptide 6	Cell motility	2	55.04	0.45	2.54E-02	1.17	1.84E-01
IPI00749113.2	DUT Isoform 3 of Deoxyuridine 5'-triphosphate nucleotidohydrolase, mitochondrial	Cycle of DNA repair	1	42.06	0.45	1.67E-01	0.57	2.32E-02
IPI00419258.4	HMGB1 High mobility group protein B1	In the nucleus interacts with nucleosomes, transcription factors and histones, organizes the DNA and regulates transcription.	5	50.23	0.44	6.41E-02	1.74	4.03E-03
IPI00410693.4	SERBP1 SERPINE1 mRNA binding protein 1, isoform CRA_d	Interact with CHD3 which is one of the components of a histone deacetylase complex	3	48.60	0.44	2.48E-04	0.64	2.95E-01
IPI00966060.1	SYNCRIP SYNCRIP protein (Fragment)	Protein synthesis	3	33.33	0.44	1.52E-02	0.26	1.77E-04
IPI00479997.4	STMN1 Stathmin	Microtubule disassembly and cell cycle	3	46.31	0.27	6.36E-03	0.51	3.94E-02
IPI00217468.3	HIST1H1B Histone H1.5	Nucleosome structure of the chromosomal fiber	9	80.09	0.26	3.88E-02	1.18	5.91E-01
IPI00217465.5	HIST1H1C Histone H1.2	Nucleosome structure of the chromosomal fiber	11	82.16	0.23	1.27E-02	1.14	5.41E-01
IPI00217466.3	HIST1H1D Histone H1.3	Nucleosome structure of the chromosomal fiber	10	77.83	0.23	2.02E-02	0.89	7.04E-01

**Table 2:** Proteins Differentially Expressed Between SKOv3 PGCCs and Control SKOv3.

**Accession**	**Protein Name**	**Function**	**Peptides (95%)**	**% Coverage**	**Ratio (Sample PGCC:control)**	**P value**
**Upregulated proteins (40)**						
IPI:IPI00923597.2	NDRG1 cDNA FLJ39243 fis, clone OCBBF2008283, highly similar to Protein NDRG1	Increase phosphorylation of NEU/ERBB2 receptor tyrosine kinase and induce the growth and differentiation of cells	5	28.29	6.49	3.94E-03
IPI:IPI00845339.1	HSPA1B;HSPA1A cDNA FLJ54392, highly similar to Heat shock 70 kDa protein 1	Important for protein folding, and help to protect cells from stress	12	45.87	5.15	2.95E-04
IPI:IPI00169383.3	PGK1 Phosphoglycerate kinase 1	Is an enzyme involved in glycolysis	11	72.66	2.49	1.36E-03
IPI:IPI00479186.7	PKM2 Isoform M2 of Pyruvate kinase isozymes M1/M2	M2-PK is a cytosolic enzyme that participates in the phosphorylation of histone 1.	18	74.39	2.43	1.40E-07
IPI:IPI00219018.7	GAPDH Glyceraldehyde-3-phosphate dehydrogenase	An enzyme related to glycolysis	15	77.31	2.33	1.51E-04
IPI:IPI00015947.5	DNAJB1 DnaJ homolog subfamily B member 1	Interact with STUB1 and HSP(heat-shock protein)A4	1	32.62	2.17	1.27E-02
IPI:IPI00302592.2	FLNA Isoform 2 of Filamin-A	Participates in the anchoring of membrane proteins for the actin cytoskeleton	19	45.62	2.16	3.50E-02
IPI:IPI00018140.3	SYNCRIP Isoform 1 of Heterogeneous nuclear ribonucleoprotein Q	Interact with ACF, APOBEC1, SYT7 and SYT9	1	26.65	2.11	4.37E-02
IPI:IPI00947127.1	LDHA L-lactate dehydrogenase A chain isoform 3	An enzyme related to glycolysis	8	59.00	2.07	1.65E-05
IPI:IPI00022774.3	VCP Transitional endoplasmic reticulum ATPase	Putative ATP-binding proteins vesicle transport and fusion	6	42.06	2.07	2.67E-03
IPI:IPI00414676.6	HSP90AB1 Heat shock protein HSP 90-beta	Molecular chaperones	8	52.35	2.06	4.19E-03
IPI:IPI00298994.6	TLN1 Talin-1	Assembly of actin filaments and spreading and migration of cells	3	30.26	2.04	2.55E-03
IPI:IPI00396485.3	EEF1A1 Elongation factor 1-alpha 1(*)	Translation and nuclear export of proteins	11	57.36	2.02	1.77E-04
IPI:IPI00218319.3	TPM3 Isoform 2 of Tropomyosin alpha-3 chain	Muscle contraction and the cytoskeleton of non-muscle cells	7	65.73	2.00	6.43E-04
IPI:IPI00900293.1	FLNB filamin-B isoform 1	Intracellular communication and signaling by cross-linking the protein actin	11	34.18	1.99	8.81E-06
IPI:IPI00465248.5	ENO1 Isoform alpha-enolase of Alpha-enolase	A glycolytic enzyme	27	84.56	1.96	9.69E-07
IPI:IPI00003865.1	HSPA8 Isoform 1 of Heat shock cognate 71 kDa protein(*)	Molecular chaperones and as an ATPase in the disassembly of clathrin-coated vesicles	14	55.57	1.86	2.00E-04
IPI:IPI00019502.3	MYH9 Isoform 1 of Myosin-9(*)	Muscle contraction and eukaryotic motility	5	38.98	1.84	1.17E-02
IPI:IPI00218918.5	ANXA1 Annexin A1	Inhibits the activation of NF-κB by binding to the p65 subunit	6	69.36	1.77	5.58E-03
IPI:IPI00909232.1	HNRNPC cDNA FLJ53542, highly similar to Heterogeneous nuclear ribonucleoproteins C	Influence pre-mRNA processing and other aspects of mRNA metabolism and transport	3	66.67	1.75	4.91E-03
IPI:IPI00930688.1	TUBA1B Tubulin alpha-1B chain	Form microtubules	12	48.12	1.74	2.23E-02
IPI:IPI00014898.3	PLEC Isoform 1 of Plectin	A link between the three main components of the cytoskeleton	3	32.71	1.71	3.68E-02
IPI:IPI00013808.1	ACTN4 Alpha-actinin-4	An actin-binding protein and involved in metastatic processes	3	21.62	1.71	3.42E-02
IPI:IPI00549725.6	PGAM1 Phosphoglycerate mutase 1	Catalyzes the internal transfer of a phosphate group	2	35.04	1.70	4.29E-02
IPI:IPI00007752.1	TUBB2C Tubulin beta-2C chain	Form microtubules	10	62.47	1.66	1.00E-02
IPI:IPI00930226.1	ACTG1 cDNA FLJ57283, highly similar to Actin, cytoplasmic 2	Maintenance of the cytoskeleton	20	70.51	1.63	8.74E-03
IPI:IPI00179330.6	RPS27A Ubiquitin-40S ribosomal protein S27a	Targeting cellular proteins for degradation	5	57.05	1.58	6.80E-03
IPI:IPI00382470.3	HSP90AA1 Isoform 2 of Heat shock protein HSP 90-alpha	Molecular chaperones	10	51.05	1.56	1.10E-02
IPI:IPI00000874.1	PRDX1 Peroxiredoxin-1(*)	A member of antioxidant enzyme	10	70.85	1.55	2.46E-04
IPI:IPI00335930.1	DAZAP1 Isoform 2 of DAZ-associated protein 1	Involved in germ cell development and gametogenesis	2	17.72	1.54	1.22E-02
IPI:IPI00010796.1	P4HB Protein disulfide-isomerase	protein disulfide isomerase	3	38.39	1.52	4.68E-03
IPI:IPI00909303.2	CTSB cDNA FLJ58073, moderately similar to Cathepsin B	Lysosomal cysteine protease	3	39.27	1.50	3.55E-03
IPI:IPI00027463.1	S100A6 Protein S100-A6	Cell cycle progression and differentiation	3	50.00	1.45	1.28E-02
IPI:IPI00418471.6	VIM Vimentin	The major cytoskeletal component of mesenchymal cells	35	81.76	1.43	1.61E-02
IPI:IPI00418169.3	ANXA2 Isoform 2 of Annexin A2	Involved in cell motility, linkage of membrane-associated protein complexes to the actin cytoskeleton, endocytosis,	11	68.35	1.41	4.26E-02
IPI:IPI00017963.1	SNRPD2 Small nuclear ribonucleoprotein Sm D2	Pre-mRNA splicing and small nuclear ribonucleoprotein biogenesis	3	60.17	1.41	3.72E-02
IPI:IPI00554648.3	KRT8 Keratin, type II cytoskeletal 8	A keratin protein and reacts mainly with secretory epithelia	13	63.35	1.35	1.43E-02
IPI:IPI00032313.1	S100A4 Protein S100-A4	Cell cycle progression and differentiation	4	57.43	1.29	4.47E-02
IPI:IPI00843975.1	EZR Ezrin	Involved in cell surface structure adhesion, migration, and organization	8	56.83	1.28	1.20E-04
IPI:IPI00003419.1	C11orf58 Small acidic protein	A kind of small acidic protein	1	26.23	1.24	4.19E-02
**Downregulated proteins (22)**						
IPI:IPI00641788.1	SNRPC U1 small nuclear ribonucleoprotein C	Maybe interact with Ewing sarcoma breakpoint region	2	40.00	0.85	1.69E-03
IPI:IPI00178440.3	SNORA41;EEF1B2 Elongation factor 1-beta	Modification of uridines to pseudouridines	4	44.44	0.76	4.01E-02
IPI:IPI00304692.1	RBMX Heterogeneous nuclear ribonucleoprotein G	An active X chromosome homolog of the Y chromosome RBMY gene	4	69.05	0.76	5.54E-04
IPI:IPI00291006.2	MDH2 Malate dehydrogenase, mitochondrial	Involved in the malate-aspartate shuttle in the metabolism	4	53.25	0.75	3.26E-02
IPI:IPI00878075.1	RANBP1 23 kDa protein	Increase GTP hydrolysis by the Ran GTPase-activating protein	3	39.00	0.75	3.04E-02
IPI:IPI00021812.2	AHNAK Neuroblast differentiation-associated protein AHNAK	Related with the pseudopod protrusion and cell migration	138	84.87	0.75	3.48E-05
IPI:IPI00647837.5	ZNF185 Isoform 3 of Zinc finger protein 185	Function as a tumor-suppressor protein by associating with the actin-cytoskeleton	5	38.41	0.74	2.01E-03
IPI:IPI00303882.2	PLIN3 Isoform B of Perilipin-3	A protective coating from the body’s natural lipases	7	62.21	0.71	2.89E-02
IPI:IPI00013894.1	STIP1 Stress-induced-phosphoprotein 1	The main function of STIP1 is to link Hsp70 and Hsp90 together.	9	45.30	0.69	2.13E-02
IPI:IPI00220362.5	HSPE1 10 kDa heat shock protein, mitochondrial(*)	Molecular chaperones	7	79.41	0.62	1.03E-04
IPI:IPI00020599.1	CALR Calreticulin	A multifunctional protein that binds Ca2+ ions, rendering it inactive	9	63.31	0.56	1.58E-03
IPI:IPI00017448.1	RPS21;LOC100291837 40S ribosomal protein S21	A kind of ribosomal protein	3	78.31	0.54	7.89E-04
IPI:IPI00419258.4	HMGB1 High mobility group protein B1	In the nucleus interacts with nucleosomes, transcription factors and histones, organizes the DNA and regulates transcription.	2	63.72	0.51	4.43E-04
IPI:IPI00012772.8	RPL8 60S ribosomal protein L8	A constituent of the elongation factor 2-binding site at the ribosomal subunit interface	1	38.91	0.49	3.11E-03
IPI:IPI00410693.4	SERBP1 SERPINE1 mRNA binding protein 1, isoform CRA_d	Interact with CHD3 which is one of the components of a histone deacetylase complex	5	43.01	0.46	1.04E-02
IPI:IPI00930174.1	HIST2H2BF histone H2B type 2-F isoform b	Nucleosome structure of the chromosomal fiber	23	79.10	0.36	4.39E-02
IPI:IPI00166293.5	HIST3H2BB Histone H2B type 3-B	Nucleosome structure of the chromosomal fiber	20	79.37	0.31	1.62E-02
IPI:IPI00215780.5	RPS19 40S ribosomal protein S19	A component of the 40S subunit	3	69.66	0.27	6.04E-03
IPI:IPI00217465.5	HIST1H1C Histone H1.2	Nucleosome structure of the chromosomal fiber	16	77.46	0.26	5.22E-03
IPI:IPI00217468.3	HIST1H1B Histone H1.5	Nucleosome structure of the chromosomal fiber	10	69.91	0.26	7.23E-03
IPI:IPI00217466.3	HIST1H1D Histone H1.3	Nucleosome structure of the chromosomal fiber	16	75.57	0.24	3.92E-04
IPI:IPI00856058.1	RPL31 60S ribosomal protein L31 isoform 3	A component of the 60S subunit	3	47.93	0.19	1.16E-02

Among the differentially regulated proteins in HEY and SKOv3 cells, the two most upregulated proteins in PGCCs were cathepsin B and cathepsin D, two proteinases known to be involved in invasion and stromal remodeling. Other upregulated proteins included those regulating apoptosis, stress response, cell motility, the nuclear envelope, and cell division. The most downregulated proteins were those involved in, cell motility, mRNA processing, and protein synthesis and modification, most notably, the histone H1 linker family of proteins that is involved in nucleosome formation. Therefore, we have validated that new proteins revealed by iTRAQ-based proteomic analysis as well as several proteins that are known to be involved in the regulation of different aspects of tumor growth may belong to key pathways involved in the regulation of PGCCs.

### Expression of stem cells and epithelial-to-mesenchymal (EMT)-related markers

 We have previously shown that hypoxia-inducible factor-1alpha (HIF-1alpha) expression is higher in CoCl_2_-induced PGCCs than in HEY or SKOv3 control cells. In this study, we examined a previously known HIF-1alpha target gene, Stanniocalcin 1 (*STC1*), which has been shown to be a secreted oncogene in ovarian cancer [22]. As expected, expression of STC1 was higher in HEY and SKOv3 PGCCs than in control cells. In addition, ELF-2alpha, a protein translation initiation factor that controls the level of protein translation, was significantly downregulated, which strongly suggests that PGCCs have low rates of protein synthesis, which is consistent with our previous findings that PGCCs are slow cycling in nature [6]. 

**Figure 2:**
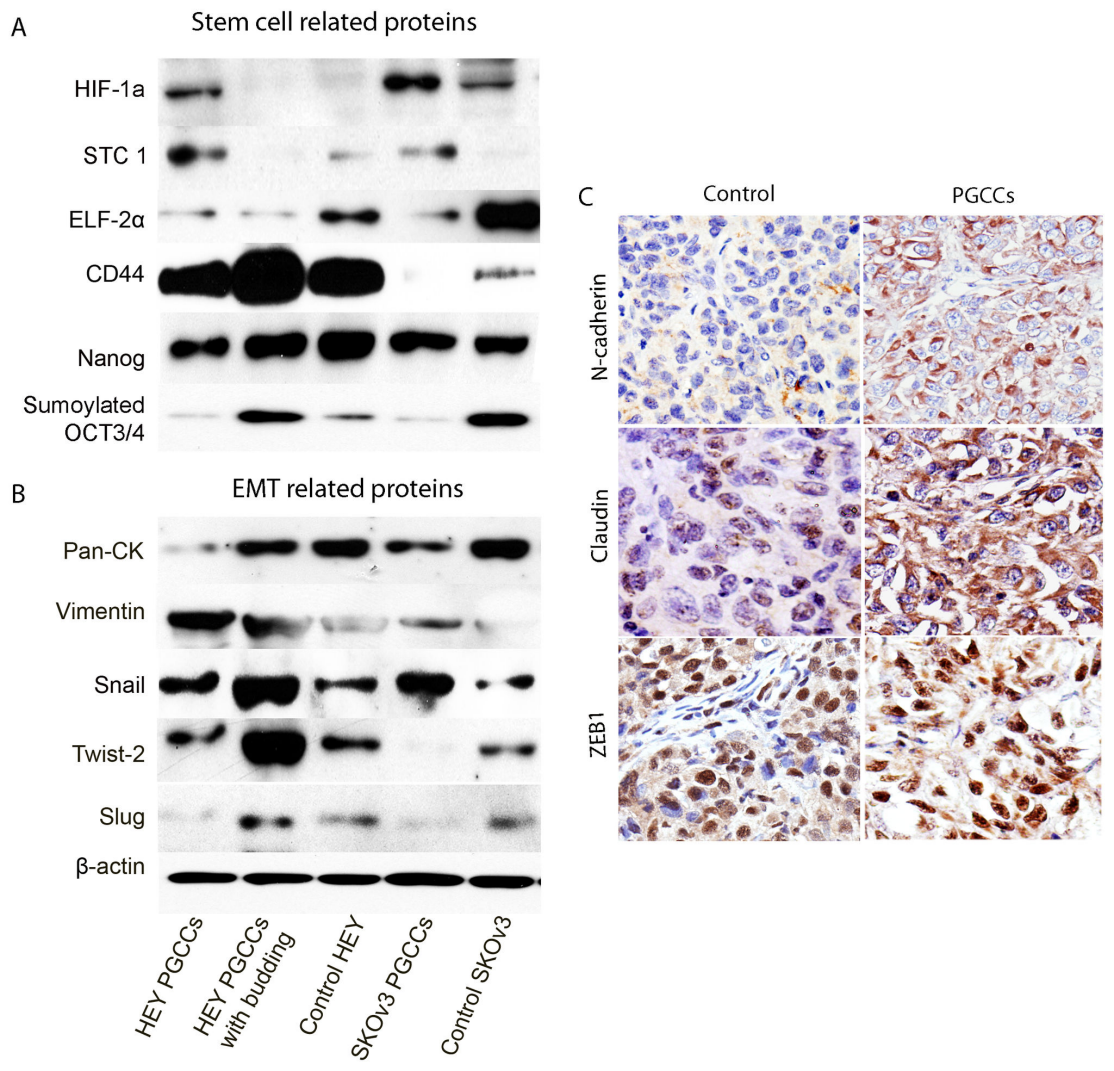
Western blot analysis of stem cell-related proteins expressed in HEY PGCCs alone, HEY PGCCs with budding daughter cells, control HEY cells, SKOv3 PGCCs alone, and control SKOv3 cells. (A) Stem cell-related protein expression. (**B**). EMT-related protein expression in HEY PGCCs alone, HEY PGCCs with budding daughter cells, control HEY cells, SKOv3 PGCCs alone, and control SKOv3 cells. (C) Immunohistochemical stain showing the EMT-related protein expression in tumor cells derived from control HEY cells and HEY PGCCs (20×).

Hypoxia has been known to promote the growth of cancer stem cells [23]. To determine whether PGCCs have increased levels of cancer stem cell markers, we examined the expression pattern of the cancer stem cell-related markers CD44, Nanog, and OCT3/4 in these cells. The highest expression of CD44 and sumoylated OCT3/4 was found in HEY PGCCs with budding daughter cells. Furthermore, control SKOv3 cells had the highest expression of CD44 and sumoylated OCT3/4 (Figure 2A).

Epithelial-to-mesenchymal transition (EMT) is known to be associated with the generation of cancer stem cell-like cells, toward this end, we examined the five samples for the expression of cytokeratin and vimentin, markers for epithelial cells and mesenchymal cells, respectively. PGCCs alone and PGCCs with budding daughter cells had lower cytokeratin expression and higher vimentin expression than control HEY and SKOv3 cells (Figure 2B), implying that the two groups of PGCCs underwent EMT. In addition, Western blot analysis demonstrated that PGCCs with budding daughter cells had the highest expression of Snail, Twist-2, and Slug (Figure 2B). The xenografted tumor cells from HEY PGCCs from our previous study [6] had higher N-cadherin and claudin expression than tumor cells derived from control HEY cells, which we confirmed via immunohistochemical staining (Figure 2C). Expression of another EMT-related protein, ZEB1, did not differ significantly in tumor cells derived from HEY PGCCs and control HEY cells. These results demonstrated that PGCCs with budding daughter cells became mesenchymal, a cancer stem cell-like phenotype. 

### Expression of DNA-interacting and protein synthesis-related proteins

**Figure 3:**
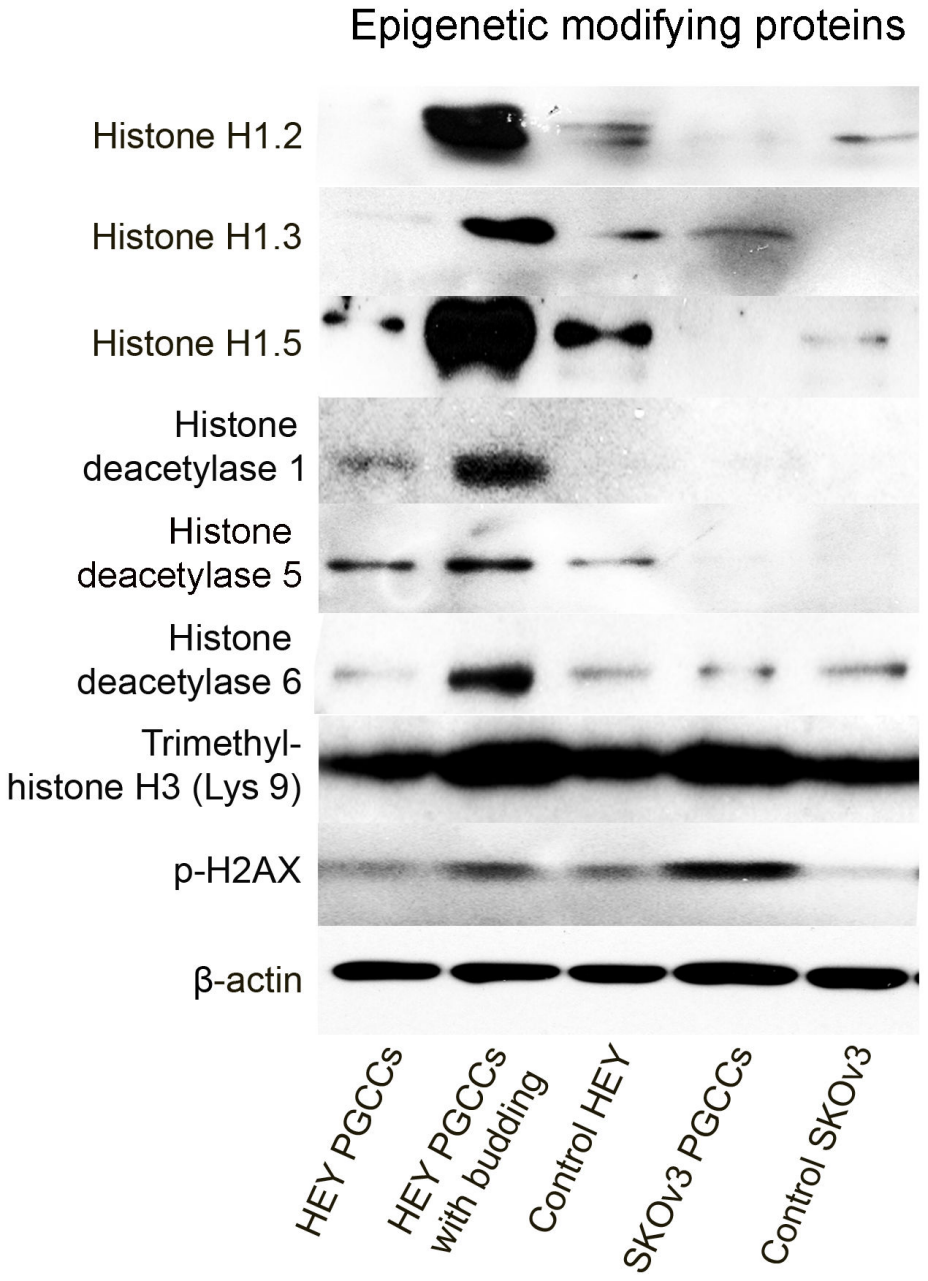
Western blot analysis of epigenetic modifying proteins in HEY PGCCs alone, HEY PGCCs with budding daughter cells, control HEY cells, SKOv3 PGCCs alone, and control SKOv3 cells.

One of most remarkable features of PGCCs is that they have multiple DNA copies, but it is unknown how these DNAs organize into nucleosomes when they encounter histones. One of most consistent changes identified through our proteomic profiling was lower levels of histone 1 in both HEY and SKOv3 PGCCs than in their respective control cells (Table 1 and Table 2), but this protein was remarkably unregulated in PGCC budding daughter cells. As shown in Figure 3, the expression of different types of histones and histone deacetylases was markedly elevated in HEY PGCCs with budding daughter cells. The expression of phosphorylated H2AX, trimethyl-histone H3, and histone 1.3 in SKOv3 PGCCs was higher than that in control SKOv3 cells (Figure 3). These results strongly suggest that histone 1-mediated epigenetic regulation may play an important role in the formation of PGCCs. 

### Dysregulation of cell cycle-related proteins

**Figure 4:**
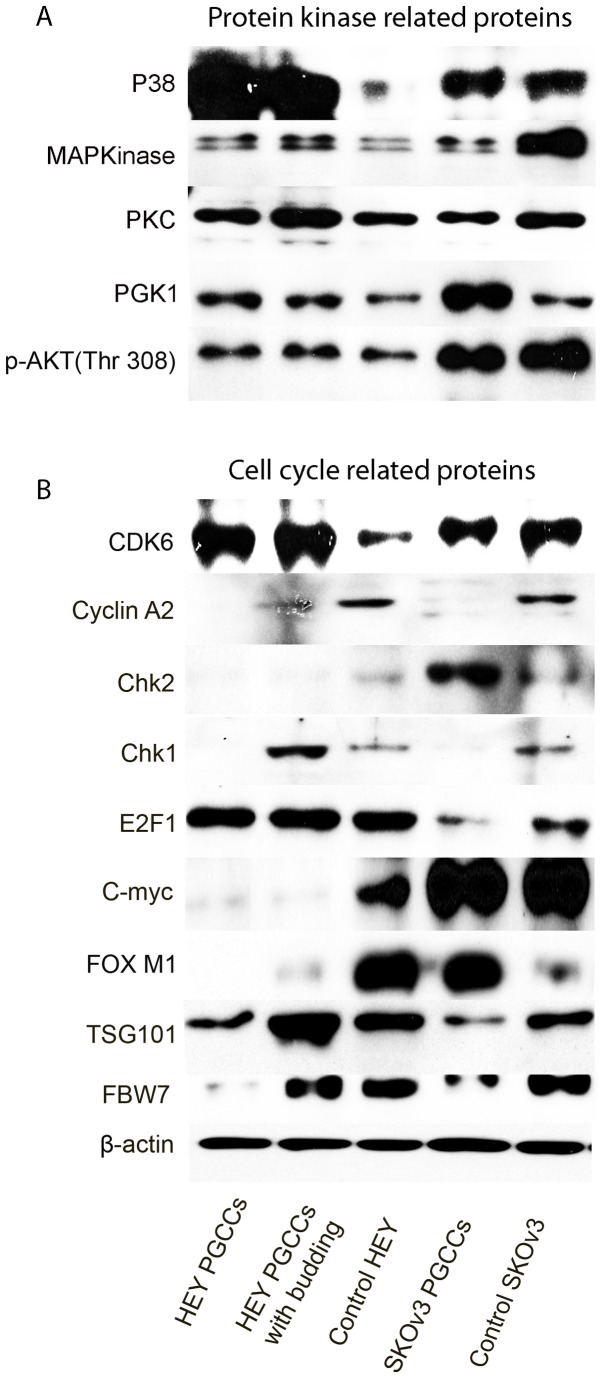
Western blot analysis of (A) kinase-related protein expression and (B) cell cycle-related protein expression in HEY PGCCs alone, HEY PGCCs with budding daughter cells, control HEY cells, SKOv3 PGCCs alone, and control SKOv3 cells.

We previously reported that formation of PGCCs involves dysregulation of cell cycle regulatory proteins [24]. The expression of protein kinases, including phosphorylated AKT (p-AKT; Thr308), protein kinase C (PKC), phosphoglycerate kinase 1 (PGK1), p38, and mitogen-activated protein kinase (MAPK), differed before and after treatment with CoCl_2_. Purified HEY PGCCs had the highest expression of p-AKT (Thr308), PGK1, and p38, whereas HEY PGCCs with budding daughter cells had the highest expression of PKC and MAPK (Figure 4A). The expression of PGK1 in SKOv3 PGCCs was higher than in control SKOv3, whereas the expression of p-AKT (Thr308), PKC, and MAPK in control SKOv3 cells was higher than in SKOv3 PGCCs.

To further examine the regulatory signature involved in the formation of PGCCs, we examined the expression of a panel of additional cell cycle-related proteins, including Chk1, Chk2, cyclin A2, CDK6, CDK2, FBW7, TSG101, FOXM1, E2F1, and c-Myc (Figure 4B). Their expression was analyzed in HEY PGCCs, HEY PGCCs with budding daughter cells, control HEY, SKOv3 PGCCs, and control SKOv3 cells. HEY PGCCs with budding daughter cells had higher levels of expression of TSG101, CDK6, and Chk1 than control HEY cells, whereas HEY PGCCs without or with budding had lower levels of c-Myc, FOXM1, FBW7, and cyclin A2 than HEY control cells. FOXM1 and Chk2 showed the opposite pattern in SKOv3 PGCCs and SKOv3 controls. 

### Expression of tumor metastasis-related proteins

**Figure 5:**
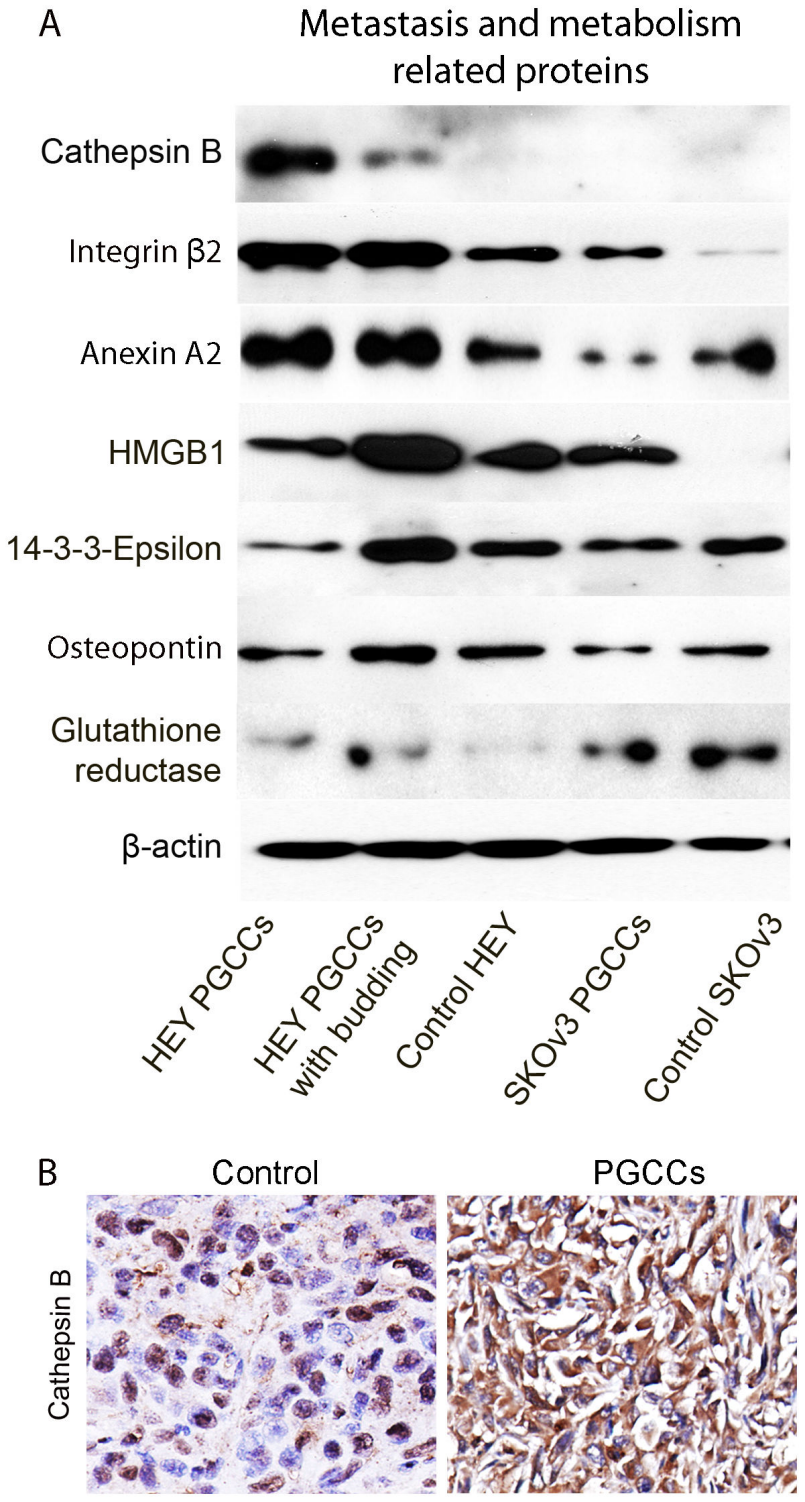
Tumor metastasis-related protein expression in HEY PGCCs alone, HEY PGCCs with budding daughter cells, control HEY cells, SKOv3 PGCCs alone, and control SKOv3 cells. (A) Western blot analysis confirming tumor metastasis-related protein expression in these cells. (B) Immunohistochemical stain of cathepsin B expression in tumor cells derived from control HEY cells and HEY PGCCs (20×).

We examined the expression of several metastasis-related proteins in PGCCs and control cells. Of these proteins, cathepsin B showed the highest expression in HEY PGCCs in our proteomic data. As expected, PGCCs alone had the highest expression of cathepsin B. Similarly, HEY PGCCs with budding daughter cells had the highest expression of osteopontin, annexin A2, HMGB1, and 14-3-3 epsilon. Expression of integrin β2 was higher in PGCCs than in control HEY and SKOv3 cells. Furthermore, expression of HMGB1 was higher in SKOv3 PGCCs than in control SKOv3 cells. Osteopontin and annexin A2 expression in control SKOv3 cells was slightly higher than in PGCCs (Figure 5A). In addition, control SKOv3 cells had increased expression levels of glutathione reductase, an enzyme related to cellular antioxidants. ElF-2α, a critical factor involved in protein translation in hypoxic microenvironments, had the highest expression in control HEY and control SKOv3 cells. Immunohistochemical staining of tumor tissue demonstrated that tumors derived from HEY PGCCs were positive for cathepsin B in the cytosol, whereas most tumor cells generated by injection of control HEY cells were positive for cathepsin B in the nucleus (Figure 5B), suggesting that formation of PGCCs may involve changes in the subcellular localization of these proteins.

## Discussion

PGCCs are generally considered to be senescent or at the stage of mitotic catastrophe, but we have shown that these large cancer cells are actually live and can generate progeny cancer cells through budding. We initially identified PGCCs after high-concentration treatment with CoCl_2_ and selective killing of regular HEY, SKOv3, and MDA-MB-231 cancer cells [6]. Studies of purified and stably passaged PGCCs showed that PGCCs can generate regular cancer cells via budding, splitting, and bursting, which are very different from the traditional concept of mitotic division for eukaryotic diploid cells. Our iTRAQ and Western blot data demonstrated that there are multiple differentially regulated proteins among PGCCs, PGCCs with budding daughter cells, and control cells for HEY and SKOv3. 

Our results identified several key signaling pathways involved in the formation of PGCCs and PGCCs with budding cells. First, hypoxia induced activation of cancer stem cells and related EMT genes. We showed that expression of HIF-1alpha and its downstream target STC1 are upregulated in PGCCs or budding cells, and this expression is associated with several cancer stem cell markers such as CD44, OCT3/4, and Nanog and several EMT-inducing transcription factors, including Twist-2 and Snail. Moreover, PGCCs may undergo different metabolic processes, including nutrient uptake and storage [24]. These factors may make PGCCs more adaptable to stress and hypoxic microenvironments than diploid cells. Second, we identified multiple epigenetic changes in PGCCs, in particular, histone 1 is down-regulated in both HEY and SKOv3 PGCCs cells but upregulated in cells that bud from PGCCs. Because PGCCs are large and have multiple copies of the genome, histones are important components of the nucleosome that can regulate DNA duplication and PGCC formation, and loss of histone 1 may be a key epigenetic event involved in the formation of PGCCs. Third, we found that alteration of several cell cycle-related proteins (Chk1, Chk2, cyclin A2, and CDK6) may be involved in the formation of PGCCs. FOXM1 protein also plays a key role in cell-cycle progression, and endogenous FOXM1 expression peaks is at the S and G2/M phases. FBW7 can bind directly to cyclin E and likely targets cyclin E for ubiquitin-mediated degradation. Other cancer-associated genes, such as Myc, can cooperate with p53 to generate polyploid cells [25]. Fourth, expression of other cell motility-related proteins, including cathepsin B, cathepsin D, 14-3-3 epsilon, HMGB1, integrin β2, and annexin A2, was increased in PGCCs with budding daughter cells. 

Although there are some common expression patterns between these two cell lines upon treatment with CoCl_2_ (such as histone 1 or STC1 expression), HEY and SKOv3 cells also display unique sets of differentially expressed proteins following the treatment of CoCl_2_. Such differences could be due to the different genetic backgrounds of these two cancer cell lines as HEY cells express wild-type p53 and SKOv3 cells have a deletion mutation of p53. The difference in proteomic expression between these two cell lines may be at least partially attributed to their p53 mutation status, and further validation with more ovarian cancer cell lines with known p53 expression patterns is warranted. 

Although mitosis prevails in complex eukaryotes, it has been well documented that variations of the mitotic cell cycle can occur in lower organisms including plants, yeasts, and viruses [24,26]. Among these variations is endoreduplication, a deviation from the normal mitotic cell cycle consisting of multiple rounds of DNA replication without an intervening mitosis step. This process is an evolutionarily conserved means of generating multinucleated cells and is common in certain types of growth in trophoblasts (to meet the high oxygen demands of fetal development) and in the generation of platelets by megakaryocytes in mammals [24,26]. In cancer patients, certain antimitotic chemotherapy drugs can increase the formation of giant cells, which are often considered to be at the stage of mitotic catastrophe and subsequent apoptosis [8]. As the PGCCs’ mechanisms bypass the time-consuming and ordered mitotic events, the PGCCs thus use each of or a combination of all of the above mechanisms from simple organisms for renewal and fast reproduction. 

Polyploidy has multiple advantages over diploidy [26-29]. For example, PGCCs are flexible and less disruptive to highly structured tissue than cells that divide via mitosis. Furthermore, PGCCs contain multiple copies of genes and have no need to segregate their chromosomes. Because PGCCs have an increased size with increased copies of the genome, they usually have alterations in the interactions of the genes, in the level of epigenetic changes and gene expression, and potentially in the amount of DNA recombination [29]. These gene expression changes are likely to be essential components of the life cycle of cancer cells and enable them to not only increase in size but also undergo highly efficient DNA replication, become increasingly resilient to hypoxia and other stresses in the tumor microenvironment, and very efficiently produce progeny cells. All of these mechanisms may work synergistically to facilitate rapid malignant growth.

## Conclusions

Our global protein analysis based on iTRAQ identified several distinct pathways for the maintenance, growth, and cell division of PGCCs that differ from those of regular diploid cancer cells. Although the biological behavior and clinical and pathological significance of PGCCs in human tumors remain to be tested, identification of protein profiles involved in PGCC formation will advance our understanding of PGCC biology and help identify possible new opportunities for targeted therapy.

## Supporting Information

Figure S1 **Pie chart showing the functional classification of iTRAQ differentially expressed proteins using the PANTHER classification in different cell types of HEY.**
(TIF)

Table S1 **Sample information and label for iTRAQ-based proteomic analysis.  **
(DOC)

Table S2 **The detailed information of HPLC gradient parameters.**
(DOC)

Table S3 **Antibodies information used in this paper.**
(DOC)
