# Supplementary figures and images for: iTRAQ-Based Proteomic Analysis of Polyploid Giant Cancer Cells and Budding Progeny Cells Reveals Several Distinct Pathways for Ovarian Cancer Development
Source: PLoS One. 2013 Nov 14;8(11):e80120. doi: 10.1371/journal.pone.0080120 (PMC3858113; doi:10.1371/journal.pone.0080120)

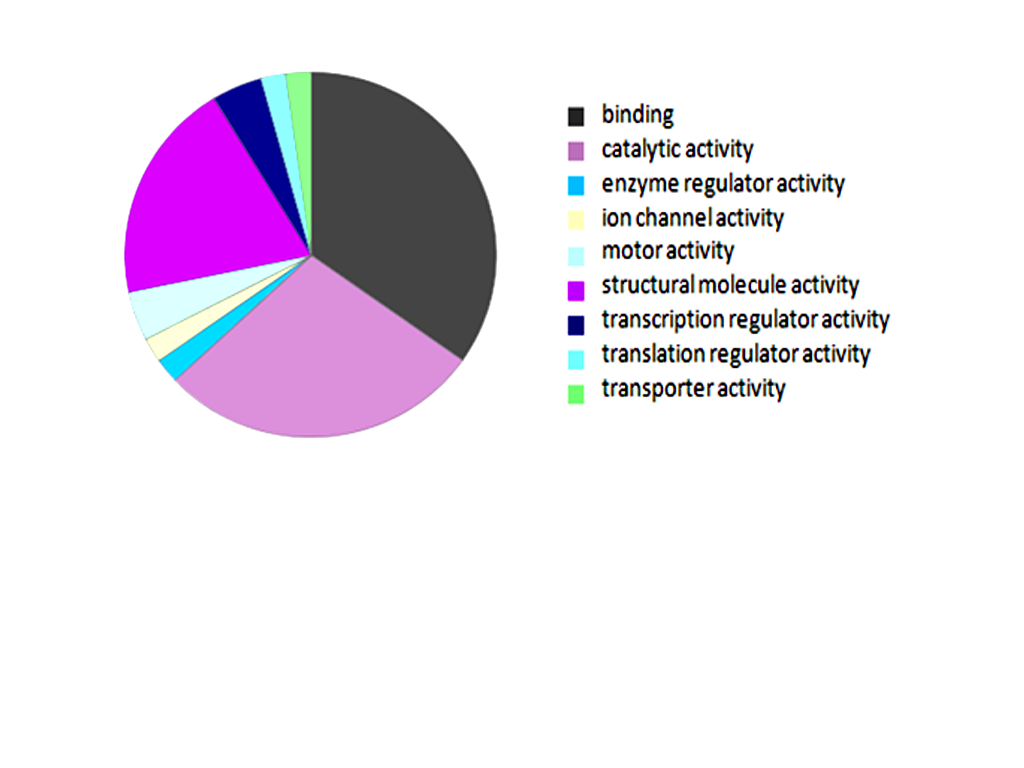

Supplement: Figure S1 — Pie chart showing the functional classification of iTRAQ differentially expressed proteins using the PANTHER classification in different cell types of HEY. (TIF) [file pone.0080120.s001.tif]
