# Supplementary material for: iTRAQ-Based Proteomic Analysis of Polyploid Giant Cancer Cells and Budding Progeny Cells Reveals Several Distinct Pathways for Ovarian Cancer Development
Source: PLoS One. 2013 Nov 14;8(11):e80120. doi: 10.1371/journal.pone.0080120 (PMC3858113; doi:10.1371/journal.pone.0080120)
Supplement: Table S1 — Sample information and label for iTRAQ-based proteomic analysis. (DOC) [file pone.0080120.s002.doc]

Supplementary table 1. Sample information and label.

| **Sample** | **Species** | **Label** |
| --- | --- | --- |
| Purified HEY PGCCs | Human | 114 |
| HEY PGCCs with budding | Human | 115 |
| Control HEY | Human | 116 |
| Purified SKOv3 PGCCs | Human | 116 |
| Control SKOv3 | Human | 117 |
| Purified SKOv3 PGCCs | Human | 114 |
| Control SKOv3 | Human | 115 |
