# Supplementary material for: iTRAQ-Based Proteomic Analysis of Polyploid Giant Cancer Cells and Budding Progeny Cells Reveals Several Distinct Pathways for Ovarian Cancer Development
Source: PLoS One. 2013 Nov 14;8(11):e80120. doi: 10.1371/journal.pone.0080120 (PMC3858113; doi:10.1371/journal.pone.0080120)
Supplement: Table S2 — The detailed information of HPLC gradient parameters. (DOC) [file pone.0080120.s003.doc]

Supplementary table 2. The detailed information of HPLC gradient parameters

| **Time / min** | **% A (0.1% Formic acid in water)** | **% B (0.1 % Formic acid in acetonitrile)** | **Flow Rate**  **(**µL **/min)** |
| --- | --- | --- | --- |
| 0.01 | 98 | 2 | 200 |
| 3 | 98 | 2 | 200 |
| 90 | 60 | 40 | 200 |
| 100 | 10 | 90 | 200 |
| 114 | 10 | 90 | 200 |
| 115 | 98 | 2 | 200 |
| 120 | 98 | 2 | 200 |
