# Supplementary material for: iTRAQ-Based Proteomic Analysis of Polyploid Giant Cancer Cells and Budding Progeny Cells Reveals Several Distinct Pathways for Ovarian Cancer Development
Source: PLoS One. 2013 Nov 14;8(11):e80120. doi: 10.1371/journal.pone.0080120 (PMC3858113; doi:10.1371/journal.pone.0080120)
Supplement: Table S3 — Antibodies information used in this paper. (DOC) [file pone.0080120.s004.doc]

Supplementary table 3. Antibodies information used in this paper

| **Antibodies name** | **Company (Cat number)** | **Dilution** | **Clone number** |
| --- | --- | --- | --- |
| Osteopontin | Abcam (ab 69498) | 1:100 (IHC) |  |
| eGFP | Santa Cruz Biot (sc-8334) | 1:700(IHC) |  |
| HIF-1α | Abcam (ab51608) | 1:1000 (WB) |  |
| EIF2α | Cell Signaling (9722) | 1:1000 (WB) |  |
| Cyclin E | Santa Cruz Biot (sc-247) | 1:500 (WB)  1:200 (IHC) | HE12 |
| Cyclin D1 | Santa Cruz Biot (sc-718) | 1:500 (WB)  1:200 (IHC) | M-20 |
| Cyclin B1 | Epitomics (1495-1) | 1:100 (IHC) |  |
| CDK6 | Santa Cruz Biot (sc-177) | 1:500 (WB)  1:200(IHC) |  |
| CDK4 | Santa Cruz Biot (sc-260 | 1:500 (WB)  1:200(IHC) | C-22 |
| CDK2 | Santa Cruz Biot (sc-163) | 1:500 (WB) | M2 |
| CD133 | Abcam (ab19898) | 1:200 (WB)  2µg/ml  1:100(IHC) |  |
| CD44 | BD Pharmigen FITC (555478) | 1:100 (IHC) |  |
| CD44 | Cell Signaling (3570S) | 1:1000 (WB) | (156-3C11) |
| β-actin | Sigma (A5316) | 1:12,000 | AC-74 |
| BRCA1 | Santa Cruz Biot (sc-642) | 1:100 (IHC) | C20 |
| BRCA2 | Genetex (GTX70121) | 1:50 (IHC) | 3E6 |
| Cathepsin B | Abcam (ab30443) | 1:1000 (WB)  1:100 (IHC) |  |
| OCT3/4 | Santa Cruz Biot (sc-5279) | 1:200 (WB)  1:100 (IHC) | C-10 |
| Human cytokeratin | Bd Biosciences (349205) | IHC only pre-diluted ready to use | CAM5.2 |
| Human Vimentin | Abcam (ab 8069) | 1:100 (IHC) | V9 |
| cytokeratin AE1/AE3 | Imgenex (IMG-80126) | 1:35(IHC)  1:350(WB) |  |
| vimentin | Neomarker(MS-129-P0) | 1:100(IHC) |  |
| vimentin | abcam (ab8978) | 1:1,000(WB) |  |
| FABP4 | abcam (ab13979) | 1:200(IHC) |  |

IHC: Immunohistochemical staining

WB: Western blot
